# Extended-spectrum-beta-lactamases and carbapenemase-producing *Klebsiella pneumoniae* isolated from fresh produce farms in different governorates of Egypt

**DOI:** 10.14202/vetworld.2022.1191-1196

**Published:** 2022-05-18

**Authors:** Esraa A. Elshafiee, Mona Kadry, Sara Mohamed Nader, Zeinab S. Ahmed

**Affiliations:** Department of Zoonoses, Faculty of Veterinary Medicine, Cairo University, Cairo, Egypt

**Keywords:** carbapenemase, extended-spectrum-beta-lactamases, fresh produce, humans, irrigation water, *Klebsiella pneumonia*

## Abstract

**Background and Aim:**

Fresh produce farms represents a major source of concern since they are becoming increasingly antibiotic resistant. This study aimed to investigate the occurrence of carbapenemase and extended-spectrum-beta-lactamases (ESBL) - producing genes in *Klebsiella pneumonia*e isolated from fresh produce farms in Egypt, irrigation water, and people working in these fields.

**Materials and Methods:**

One hundred tomatoes from typical farms were collected in plastic bags. The study also included 20 surface-water samples from different irrigation watersheds in fresh produce farms, as well as 50 feces samples from farmworkers. Suspected *K. pneumoniae* was grown on Eosin Methylene Blue agar for 24 h before being biochemically identified using the RapID ONE test. PCR was used to detect carbapenemase (*bla*KPC, *bla*OXA-48, and *bla*NDM) and ESBL (*bla*SHV, *bla*TEM, and *bla*CTX) expressing genes on isolates.

**Results:**

*K*. *pneumoniae* was identified in 30% of water and 10% of worker samples, while only one isolate was found in tomato samples. One of the six irrigation water isolates tested positive for carbapenem-resistant NDM. In contrast, two isolates tested positive for ESBL determinants, one of which was *bl*SHV and the other having both *bla*SHV and *bla*TEM genes. Two of the five *K. pneumoniae* isolates from farmworkers were positive for *bla*NDM, with one isolate also testing positive for *bla*SHV and *bla*TEM. The *bla*OXA-48 gene was also discovered in the carbapenem-resistant *K. pneumoniae* tomato isolate used in this study.

**Conclusion:**

Carbapenemase- and ESBL-producing *K. pneumoniae* were found in fresh produce farms, implying that these resistance genes were being passed down to Egyptian consumers.

## Introduction

On the farm, during the pre-harvest, harvest, and post-harvest operations and the transportation and processing lines, fresh food might be infected with hazardous enteric bacteria. Two of the most prevalent sources of pollution are untreated wastewater and animal/human excrement [1,2]. In the commonly recorded epidemics of gastrointestinal sickness (mostly in association with the Enterobacteriaceae family), fresh fruits were shown to be the source of bacterial contamination, and *Klebsiella* *pneumoniae* was identified as a prominent food-borne pathogen [3]. Irrigation systems, as well as post-harvest vegetable washing, usually employ untreated surface water or microbe-contaminated groundwater. Consumers may be exposed to health concerns if the water contains dangerous microbes. As a result, vegetable consumption’s promise of nutrition and health benefits could be a source of infection for many infectious diseases [4].

According to Pachepsky *et al*. [5], many cases of gastrointestinal diseases caused by interactions with irrigation water-related microbial contamination that was passively associated with vegetable handling go unreported. As a result, it highlights the importance of gathering additional scientific evidence tying sickness to direct food or water consumption. Antibiotic resistance in both saprophytic and pathogenic microorganisms in fresh vegetables may have a role in bacterial population horizontal resistance distribution. Meaning that fresh produce may serve as a transporter and reservoir for antibiotic-resistant bacteria [6,7]. *K. pneumoniae* is an opportunistic bacterium that led to a wide-ranging of illnesses in both the community and the hospital [8]. It mostly affects disabled or immunocompromised patients, causing urinary tract infections and pneumonia [9]. Due to the high frequency of isolates with multidrug-resistance, *K. pneumoniae* poses significant therapeutic challenges, leaving only a limited number of therapeutic alternatives.

In Gram-negative bacteria, *K. pneumoniae*, has a variety of approaches to combat the effects of antibiotics, including the enzymes production, changes in antibiotic cellular permeability and reductions in antibiotic cellular permeability, as well as modification of the bacterial membrane by decreasing porin synthesis or increasing the expression of efflux pump systems [10]. These occurrences have been observed in several clinical isolates with a multidrug-resistant phenotype [11].

*bla*TEM genes, *bla*SHV genes, and, in particular, *bla*CTX-M genes are among the most commonly mentioned either extended-spectrum-beta-lactamases (ESBL) genes. In 2020 [12], the selection of resistant mutants by the overproduction of chromosomal AmpC by the acquisition of either ESBLs or plasmidic AmpC (pAmpC) has limited the use of third-generation cephalosporins for the treatment of severe Gram-negative bacterial infections [13]. However, in *K. pneumoniae*, carbapenem resistance is mediated by a number of mechanisms, the most famous of which is the production of carbapenemase enzymes (e.g., *bla*KPC, *bla*NDM, *bla*VIM, and *bla*OXA-48) [14].

Antimicrobial-resistant bacteria may be able to spread to fresh produce by contaminating irrigation, water, and manure sprayed onto agricultural fields [15]. Resistant bacteria can colonize fresh fruits and vegetables in a variety of methods, involving the use of antibiotics directly in the cultivation process and irrigation with contaminated water, which represents a public health concern.

Although the significance of food in human contact with antibiotic-resistant bacteria is yet unknown, it is becoming a rising food safety concern. Ingested *K. pneumoniae* strains may carry ESBL and pAmpC genes transported by mobile genetic elements, colonizing the human commensal flora.

The use of significant amounts of antibiotics in plant agriculture, as well as the application of manure from animal farming to agricultural fields, is the most common origins of antibiotic resistance. However, hospitals and commercial animal husbandry are the most common sources that have contributed to the selection of resistant bacteria in plants. As a result, this study aimed to look for evidence of ESBL- and carbapenemase-producing genes in *K. pneumoniae* isolated from Egyptian fresh produce farms.

## Materials and Methods

### Ethical approval

The study protocol was reviewed and approved by the local guidance of the Research Ethics Committee of the Faculty of Veterinary Medicine, Cairo University, Egypt.

### Study period and location

This study was conducted from February to July 2019 in different farms from Cairo, Giza, Fayoum, and Ismailia governorates of Egypt.

### Sample collection

In sterile 250-mL polyethylene bottles, a number of 20 surface-water samples were collected from fresh produce farms from Cairo, Giza, Fayoum, and Ismailia governorates of Egypt.

including different irrigation watersheds. In addition, 100 tomatoes were collected in plastic bags from the same traditional farms. Each sample’s information (farm address, date, and irrigation type) was recorded. Fifty farmers who work on the farms provided feces samples. All of the samples were submitted to the lab in an icebox at 4°C.

### Bacterial isolation and identification

Filtration of all water samples was done on arrival using the membrane filtration technique, as recommended by the American Public Health Association [16]. One hundred milliliters of water samples were passed through a vacuum pump filter device with a sterile filter membrane having a pore size of 0.45 m that contained microorganisms in this method. After filtration, the bacteria-containing membranes were spread over Oxoid’s Eosin Methylene Blue (EMB) Agar and incubated at 37°C for 24 h.

Tomato samples were sterilized on the surface by immersing them in a 70% ethanol solution and drying them. Alegbeleye Guo *et al*. [17] indicated that the tomatoes were sliced into stem scar and pulp using a sterile knife. Each sample was stomached in a sterile stomacher bag with 9 mL of sterile 0.1% peptone water for 2 min. One milliliter of the sample was mixed with 9 mL of tryptic soya broth (TSB) and incubated at 37°C for 24 h.

After that, a loop of tryptic soya enrichment was streaked onto EMB agar, which was then incubated at 37°C for another 24 h. Suspected *K. pneumoniae* colonies were sub-cultured onto nutrient agar plates and provisionally identified using morphological characteristics, Gram’s stain, and biochemical characteristics [18]. The RapID ONE test was used to identify *K. pneumoniae* (Oxoid-remel USA) biochemically.

### PCR for the detection of carbapenemase and ESBL-encoding genes

For the molecular detection of carbapenemase and ESBL-encoding genes, the DNA of *K. pneumoniae* strains was extracted using a QIAamp1 Mini Kit (Qiagen, Hombrechtikon, Switzerland. There were specific oligonucleotide primers for multiplex PCR for ESBL-encoding genes (*bla*CTX-M, *bla*SHV, and *bla*TEM) and carbapenemase-encoding genes (*bla*KPC, *bla*OXA, and *bla*NDM).

## Results and Discussion

Antimicrobial resistance has emerged as one of the most serious threats to human and veterinary medicine worldwide. ESBLs and carbapenemase-producing Gram-negative bacteria, particularly *K. pneumoniae*, have recently emerged as major pathogens in both hospital-acquired and community-acquired illnesses worldwide. However, information on the role of fresh produce (which is usually consumed raw) in the exposure of human beings to such germs is still limited.

Result revealed from using primer sequences for PCR amplification as shown in Table-1 [19] that *K. pneumoniae* was found in high concentrations (30%) in irrigation water (Table-2). Podschun *et al*. [20] reported similar results, finding *K. pneumoniae* in 31.6% of freshwater samples. Hamza *et al*. [12] found *K. pneumoniae* in a fish pond water intake recovered from farm drainage in Egypt, with a prevalence of 13.3%. This variation in the occurrence rate could be because the irrigation watersheds we studied were near a wide range of agricultural activities, such as the production of livestock, dairy, and poultry farm waste, which can serve as reservoirs for many pathogenic bacteria that can contaminate the plantation environment and, consequently, surface waters [21].

**Table 1:** Primer sequences used for PCR amplification of extended-spectrum-beta-lactamases and carbapenemase-encoding genes.

Target gene	Primer sequence (5′-3′)	Amplified length (bp)	Reference
*bla* KPC	ATG TCA CTG TAT CGC CGT CT (F) TTT TCA GAG CCT TAC TGC CC (R)	882	[19]
*bla* OXA-48	TTG GTG GCA TCG ATT ATC GG (F) GAG CAC TTC TTT TGT GAT GGC (R)	743	
*bla* NDM	GGT TTG GCG ATC TGG TTT TC (F) CGG AAT GGC TCA TCA CGA TC (R)	621	
*bla* CTX-M	GCGATGGGCAGTACCAGTAA (F) TTACCCAGCGTCAGATTCCG (R)	392	
*bla* TEM	ATGAGTATTCAACATTTCCG (F) TTACCAATGCTTAATCAGTGAG (R)	861	
*bla* SHV	TCAGCGAAAAACACCTTG (F) TCCCGCAGATAAATCACCA (R)	472	

**Table 2:** Occurrence of ESBL- and carbapenemase-producing *K. pneumoniae* in tomatoes, irrigation water, and farmworkers at five fresh produce farms in Giza Governorate, Egypt.

Source of samples	No. of samples examined	*K. pneumoniae* positive samples No. (%)	ESBL-producing *K. pneumoniae* No. (%)	Carbapenemase-producing *K. pneumoniae* No. (%)	Both
Tomatoes	100	1 (1)	0 (0)	1 (100)	0 (0)
Irrigation water	20	6 (30)	2 (33.3)	1 (16.6)	1 (16.6)
Farmworkers	50	5 (10)	1 (20)	2 (40)	0 (0)
Total	170	12 (7.1)	3 (25)	4 (33.3)	1 (8.3)

ESBL=Extended-spectrum-beta-lactamases, *K. pneumonia=Klebsiella pneumoniae*

Concerning irrigation water samples, *K. pneumoniae* determinants revealed that two strains are ESBL producers, with two genotypes (*bla*SHV and *bla*TEM) carried on the identified isolates; however, only one carbapenem-resistant *K. pneumoniae* carrying *bla*NDM was found (Tables-3 and 4). For infections caused by ESBL-producing bacteria, carbapenems are the antibiotics of choice. Unfortunately, carbapenem use has been associated with the growth of carbapenem-resistant bacteria such as *Klebsiella*. Poor-quality water can be a direct source of contamination and a vehicle for spreading localized contamination in the field, facility, or transportation environments when growing fresh produce crops.

**Table 3:** Pattern of genotypic β-lactamase- and carbapenemase-encoding genes in six *Klebsiella pneumoniae* isolates.

Source of isolates	β-lactamases genes	Carbapenemase genes
Tomato	0	OXA-48
Irrigation water	SHV	NDM
Irrigation water	SHV, TEM	0
Farmworker	0	NDM
Farmworker	SHV, TEM	0
Farmworker	0	NDM

**Table 4:** Occurrence of β-lactamase- and carbapenemase-encoding genes in six *K. pneumoniae* isolates.

*K. pneumonia* isolates (n)	Resistant genes							

	Carbapenemase determinants				Extended-spectrum-beta-lactamases determinants			
	
	No.	NDM	OXA	KPC	No.	SHV	TEM	CTX
Tomatoes (1)	1	0	1	0	0	0	0	0
Irrigation water (6)	1	1	0	0	2	2	1	0
Farmworkers (5)	2	2	0	0	1	1	1	0
Total	4	3	1	0	3	3	2	0

K. pneumonia=Klebsiella pneumonia

*K. pneumoniae* has been identified in carrots, arugula, iceberg lettuce, cucumber, tomato, and sprouts, among other vegetables [22]. Unlike water, just one isolate of *K. pneumoniae* was found in the tomatoes that were picked (Table-2). This is lower than the results obtained by Puspanadan *et al*. [23], who found 13% (20) *K. pneumoniae* in tomato samples. The main sources of contamination are fertilizers made from animal waste, polluted irrigation water, and post-harvest washing with contaminated water. Because some farmers utilize animal dung or excrement as a fertilizer, the upper layer of soil (30 cm2 above the ground) often contains 106-107 bacteria/g. On the other hand, contamination might arise as a result of systemic contamination that begins at the cultivation site and continues through storage and handling. African studies on this topic are extremely rare.

Although our tomato samples were cultivated in a region where the pathogen is common in irrigation water sources, the low prevalence of *K. pneumoniae* in our tomato samples could be due to the analysis methods, which rely on rinse operations to remove target microorganisms. This approach, which avoids post-harvest rinse and disinfection, was designed to retrieve internalized bacteria that could constitute a public health danger [24]. As a result, tomato samples were prepared for the current investigation by sterilizing the surface and homogenizing the internal tissue.

This discovery also underscores the risk of illness from eating raw tomatoes, which are commonly taken without being cooked. Furthermore, they may serve as a direct source of carbapenemase-producing bacteria in the colon, facilitating the spread of carbapenemase genes to commensal microflora, which then passes them on to other pathogenic microorganisms [18].

The presence of OXA-48 type carbapenemases in tomato samples was discovered in our study (Tables-3 and 4). To the best of our knowledge, this is the first report of OXA-48-like generating *K. pneumoniae* being recovered from fresh vegetables in Egypt. Carbapenemases of the OXA-48 type have been found in several North African and other Mediterranean countries [25,26].

*K. pneumoniae* has been found to be connected with mobile genetic elements such as plasmids or transposons, allowing them to spread more easily in the community and environment [27]. This is especially important because most agricultural products are ready-to-eat, which implies that, unlike food from animals, the customer will be directly exposed to resistant germs because hygienic methods like boiling do not deactivate them.

Because of the limited therapeutic choices and higher morbidity and mortality associated with this resistant strain, it is considered a major public health concern [28]. ESBL-producing *K. pneumoniae*, on the other hand, poses a threat to a variety of ecological niches, including animals, food items, soil, and wastewater. In fact, contact with the blood, saliva, feces, and urine of ESBL-carrier animals, or the intake of contaminated water or food products, can cause people to become colonized or infected with ESBL-producing *K. pneumonia*.

According to the findings of the current investigation, the prevalence of *K. pneumoniae* in farmworkers was 10% (Table-2), which is similar to the findings of Founou *et al*. [29], who isolated *K. pneumoniae* from 11.26% of exposed workers in Cameroon. Abo Samra *et al*. [30] found that *K*. *pneumoniae* was present in 143 (6.94%) of the isolates. The difference in Klebsiella prevalence between the studies could be due to the clinical state of the patients and the sample size used in the study.

The results of ESBL and carbapenemase-resistant genes in humans revealed that out of the total of five *K. pneumoniae* strains, two were carbapenemase-positive, including *bla*NDM, and one contained beta-lactamase-generating genes, including *bla*TEM and *bla*SHV in Tables-3 and 4.

Because of the scarcity of studies on human health, animal health, and the food chain on the continent, the true prevalence of ESBL in Africa is unknown and likely underestimated. Nonetheless, some investigations on the continent have validated the global spread and high prevalence of ESBL-producing *K. pneumoniae*, although focused on human health and ignoring animal health [31-35]. A multicenter study, for example, discovered ESBL-producing *K. pneumonia* with a prevalence of 9-16% in public hospitals in Abidjan, Casablanca, Yaoundé, and Antananarivo. During a national sentinel site surveillance of resistant *K. pneumoniae*, Perovic *et al*. [36] reported a 68.3% prevalence of ESBL-producing *K. pneumoniae* in clinical samples from 2010 to 2012. Founou *et al*. [29] reported a 22.15% prevalence of fecal ESBL-producing *K. pneumoniae* carriage in Ngaoundere, Cameroon.

Antibiotics are widely used in agriculture and animal livestock production, resulting in the selection of resistant bacteria, particularly in the gastrointestinal tracts of animals; both resistant bacteria and non-absorbed antibiotics are excreted by animals in manure and waste-holding systems [37-40].

Furthermore, the use of contaminated irrigation water allows antibiotics and genetic resistance determinants to enter the environment, contaminating the soil and plants. This raises concerns about environmental pollution and the transfer of antibiotic-resistant bacteria through livestock manure. To support proper management processes for the protection of fruit and vegetable growers, farmworkers, and customers, the surveillance of irrigation water and water used to wash fresh produce is required.

Fruits and vegetables can be contaminated during the preharvest, harvest, and post-harvest stages of the farm-to-table cycle. The contamination of fruit and vegetable plants/trees during harvesting can be caused by manure treatment, irrigation with contaminated sewage water, cultivation in locations with high levels of pathogenic microorganisms, and so on [41].

Environmental isolates of drug-resistant bacteria can be a source of resistance genes for pathogenic bacterial strains, representing a severe food safety risk. Antibiotic overuse in agricultural and animal husbandry has resulted in the selection of drug-resistant bacteria [42].

## Conclusion

The presence of ESBL and carbapenemase-resistant genes in fresh food is a sign that antibiotic resistance is spreading in the environment and they can be passed down the food chain from humans to animals and from animals to humans. Once ingested, these could spread resistance to gut microflora and, eventually, dangerous germs. It is consequently critical to limit the irrational use of antibiotics in humans, as well as in animal husbandry and agriculture. To prevent the bidirectional transmission of the encoding genes, continuous surveillance of resistance to these “last resort” medicines is essential. Further studies are required in comparative genomic analysis, including genome sequencing, to recognize resistance gene profiles and their epidemiological sources in *K*. *Pneumoniae* strains isolated from fresh produce.

## Authors’ Contributions

EAE and ZSA: Designed the study and performed the methodology and investigation. MK: Drafted and revised the manuscript. SMN: Data analysis. All authors have read and approved the final manuscript.
